# *Staphylococcus aureus* in a neonatal care center: methicillin-susceptible strains should be a main concern

**DOI:** 10.1186/2047-2994-3-21

**Published:** 2014-07-01

**Authors:** Sara Romano-Bertrand, Anne Filleron, Renaud Mesnage, Anne Lotthé, Marie Noëlle Didelot, Lydie Burgel, Estelle Jumas Bilak, Gilles Cambonie, Sylvie Parer

**Affiliations:** 1 Université Montpellier 1, UMR 5119, Equipe Pathogènes et Environnements, U.F.R. des Sciences Pharmaceutiques et Biologiques, 15, Avenue Charles Flahault, BP 14491, 34093 Montpellier Cedex 5, France; 2 Centre Hospitalier Régional Universitaire de Montpellier, Hôpital La Colombière, Service d’Hygiène Hospitalière, 39 avenue Charles Flahault, 34295 Montpellier Cedex 5, France; 3 Centre Hospitalier Régional Universitaire de Nîmes, Service de Pédiatrie, Hôpital Caremeau, Place du Pr R. Debré, 30029 Nîmes Cedex 9, France; 4 Centre Hospitalier Régional Universitaire de Montpellier, Service de Néonatologie, Hôpital Arnaud de Villeneuve, 371 Avenue du Doyen Gaston Giraud, 34295 Montpellier Cedex 5, France; 5 Centre Hospitalier Régional Universitaire de Montpellier, Laboratoire de Bactériologie, Hôpital Arnaud de Villeneuve, 371 Avenue du Doyen Gaston Giraud, 34295 Montpellier Cedex 5, France

**Keywords:** *Staphylococcus aureus*, Prevalence, Catheter-related infections, White coat contamination, Methicillin-susceptible *Staphylococcus aureus*

## Abstract

**Background:**

In the context of a methicillin-susceptible *Staphylococcus aureus* (MSSA) outbreak, we aimed to improve our knowledge of *S. aureus* (SA) epidemiology in the neonatal care center (NCC) of a tertiary care teaching hospital.

**Methods:**

We performed a complete one-year review of SA carrier, colonized or infected patients. Monthly prevalence and incidence of SA intestinal carriage, colonization and infection were calculated and the types of infection analysed. During the MSSA outbreak, strains were studied for antimicrobial resistance, content of virulence genes and comparative fingerprint in Pulsed-Field Gel Electrophoresis. Hand hygiene and catheter-related practices were assessed by direct observational audits. Environmental investigation was performed in search of a SA reservoir.

**Results:**

Epidemiological analyses showed 2 or 3 prevalence peaks on a background of SA endemicity. In the NCC, during 2009, overall MSSA prevalence did not decrease below 5.5%, while mean MRSA prevalence was about 1.53%. Analysis of infection cases revealed that the outbreak corresponded to the emergence of catheter-related infections and was probably related to the relaxation in infection control practices in a context of high colonization pressure. Health care workers’ white coats appeared as a potential environmental reservoir that could perpetuate SA circulation in the ward.

**Conclusion:**

This report emphasizes the importance of integrating MSSA along with methicillin-resistant SA in a program of epidemiological surveillance in the NCC.

## Background

The high incidence of hospital-acquired infections (HAIs) in neonatal intensive care units (NICUs) is related to the immaturity of patients who are also subjected to many invasive procedures. Coagulase negative staphylococci (CoNS) and *Staphylococcus aureus* (SA) are the main and often sole bacteria colonizing the digestive tract of low birth-weight infants during the 3 first weeks of life [1]. Furthermore, CoNS and SA are responsible for most infections in hospitalized preterm infants [2-4].

As in other hospital units, methicillin-resistant *S. aureus* (MRSA) outbreaks have often been reported in NICUs [5-8] whereas, at first glance, methicillin-susceptible *S. aureus* (MSSA) outbreaks seem less frequent. Indeed, a PubMed database search returns 1108 papers for “MRSA outbreak” versus 52 for “MSSA outbreak” (January 2014). In second analysis, the scarcity of MSSA outbreaks could be due to a bias in detection or reporting, MRSA being one of the most threatening pathogens as well as the principal indicator of nosocomial risk. Patients’ screening and outbreak alert systems in most hospitals focus on MRSA, while MSSA infections are generally treated piecemeal with little or no insight into molecular typing and epidemiology. However, a study of 358 *S. aureus* strains (2,007,681 days of hospitalization in 32 healthcare institutions) showed there is a significant increase of bloodstream HAIs largely due to MSSA strains [9,10].

The published MSSA outbreaks concerned merely NICUs [11-13], burns units [14] and multi-resistant MSSA [14,15]. The spread of MSSA clonal strains in NICUs seems to be very successful. For instance, in a 5-year outbreak affecting 202 neonates, classical control measures failed to end the outbreak [13] but atypical reservoirs near the patient, such as skin protectant [13] and ultrasound gel [12] were found. Intestinal carriage of SA seems to be neglected in NICUs, although it frequently occurs in infants [1,16,17]. Furthermore, it is associated with a high risk of skin colonization which can in turn increase the risk of infections, environmental contamination and cross transmission [17].

The aim of this study is to describe the epidemiology of SA in the neonatal care center (NCC) of the Montpellier Academic Hospital. An increase of MSSA colonization and infection cases in the NCC led to a complete investigation: (i) analysis of cases, (ii) assessment of hygiene practices (hand hygiene and catheter-related care) (iii) search of SA environmental reservoir, (iv) molecular typing. The outbreak was confirmed and related to the spread of a common strain with a probable environmental reservoir.

## Methods

### Settings, patients and infection surveillance policies

The NCC of Montpellier is organized in 3 sectors: the paediatric reanimation and intensive care unit or PRICU (14 beds in 9 boxes for neonates including very preterm neonates and 6 beds in 5 rooms for infants), the NICU (24 beds in 10 boxes), and a mother-cum-child or “kangaroo” ward (9 beds in individual rooms, and 3 beds in a nursery).

Hospitalized patients in the PRICU are low birth-weight (<1500 g) preterm infants and newborns aged < 1 month with diseases or unstable states. The PRICU also hosts newborns having surgery and older children requiring intensive care. Newborns are transferred to the NICU once their clinical state is stabilised or improved. As soon as their condition allows, patients are transferred to the kangaroo ward before returning home.

The medical and paramedical teams from the PRICU, the NICU and the kangaroo ward, a microbiologist and a member of the infection control (IC) team meet weekly to discuss HAI cases, differentiating true HAIs from bacterial carriage or colonization by confronting microbiological data, biological and clinical contexts and initiation of antimicrobial treatment [18]. Infants are considered as colonized if a positive culture is obtained from a non-sterile site, and infected if a pathogen is isolated from a normally sterile site or if cultures are obtained for clinical purposes. In addition, in the NICU sector, the digestive carriage of SA and multi-drug resistant bacteria (MDRB) is screened for each patient upon admission and once a week thereafter. A positive culture from a digestive sample is considered as SA carriage [17]. Stool samples are cultured to determine methicillin and ceftazidime resistance in staphylococci and gram-negative bacilli, respectively. MDRB detection leads to supplementary infection control measures to prevent cross-contamination among patients, and to further environmental investigations. A real-time surveillance is implemented by a daily account of all newly MDRB colonized or infected patients by the microbiologist, using an antibiotic resistance information system (Sirweb®, i2a, Montpellier, France). Surveillance data is transmitted to the IC team via the hospital information system. This automatic surveillance system operates for MDRB only; hence outbreaks involving susceptible micro-organisms can be detected only through clinical observation of an increased number of cases over a given time span.

### Outbreak investigation of MSSA infections

This clinical surveillance thus detected an increase in HAIs involving MSSA, leading to an outbreak investigation by the IC team including analysis of patients’ medical records, environmental investigation, and assessment of healthcare practices. Clinical strains were tested for their antimicrobial susceptibility by disk diffusion assay according to the French Committee for Antimicrobial Susceptibility Testing (Members of the Société Française de Microbiologie Committee, 2003). After extraction of the DNA performed according to Predari *et al*. [19], isolates were screened for genes encoding staphylococcal enterotoxins A (*sea*), toxic shock syndrome toxin 1 (*tst*), Panton-Valentine leukocidin (PVL; *luk- PV*) [2-4]. Clinical MSSA strains were explored by genomotyping to determine genetic links between them. For this purpose, intact genomic DNA was extracted in agarose plugs and digested by the endonuclease *Sma*I, as described [20]. Macrorestriction fragments were separated by pulsed-field gel electophoresis (PFGE) by a ramp of pulses of 20s to 5 s at 6 V/min during 24 h on CHEF-DRII apparatus (Biorad).

Dry and humid surfaces were sampled, as well as health-care workers’ clothes (i.e.: white over-gowns used for the manipulation of central venous lines, especially the front part which comes into contact with patients); the cotton swabs used for sampling served to inoculate trypticase-soy, Chapman and McConkey agar plates (bioMérieux, France) which were incubated at 37°C for 48 h. Water was sampled at different points of use in the units in 250 mL sterile bottles containing sodium thiosulfate for chloride inhibition. Water samples were passed through a 0.22 μm nylon filters, which were then incubated at 30°C during 48 h on Chapman agar. Healthcare practices in the NCC were audited by direct observation of hand hygiene opportunities (a minimum of 30 observations) and central venous catheter manipulations (a minimum of 5 observations).

### Epidemiology

Monthly prevalence and incidence of SA colonizations and infections were calculated for each sector, as well as the prevalence of MSSA and MRSA for all the NCC. Prevalence was defined as number of patients with SA *per* total number of hospitalized patients in the month, reflecting the endemicity of SA in the NCC. The incidence was the ratio of new cases on new admissions during the month. Duplicate cases in a same sector (repeat admission) or between sectors (transfer of a previously known case) were excluded from prevalence and incidence calculations.

### Ethical considerations

We studied bacterial isolates obtained during the daily care of preterm infants in our NICU. Therefore, this observational study was fully in line with the routine care of preterm infants and did not require the agreement of the ethical committee of our institution.

## Results

### SA epidemiology in the NCC

**Figure 1:**
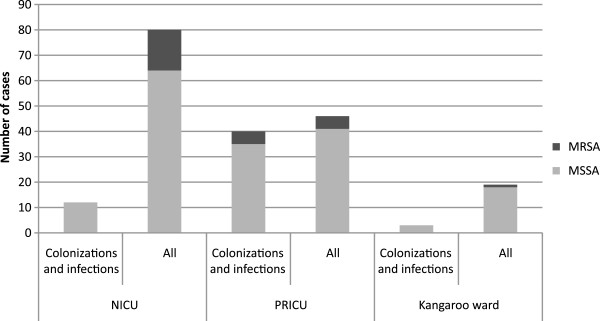
**Distribution of SA carriage, colonisation and infection for each sector in 2009.** All = carriage, colonization and infection.

One hundred and thirty nine patients admitted to the NCC were included in the epidemiological study. Distribution of carriage, colonization and infection cases for each sector is summarized in Figure 1. Fifty-four patients were carriers, colonized or infected by SA, 22 in the summer period (4, 6, 4 and 8 respectively in June, July, August and September). In the NICU and kangaroo ward, most SA isolates were involved in carriage with a low frequency of colonization and/or infection. By contrast, patients in the PRICU with more instable clinical status were more frequently colonized and/or infected.

**Figure 2:**
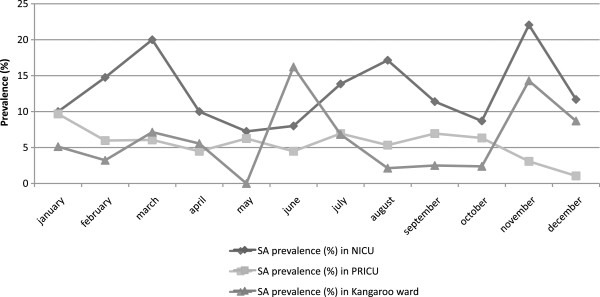
Monthly prevalence of SA carriage, colonization and infections by care sector in 2009.

**Figure 3:**
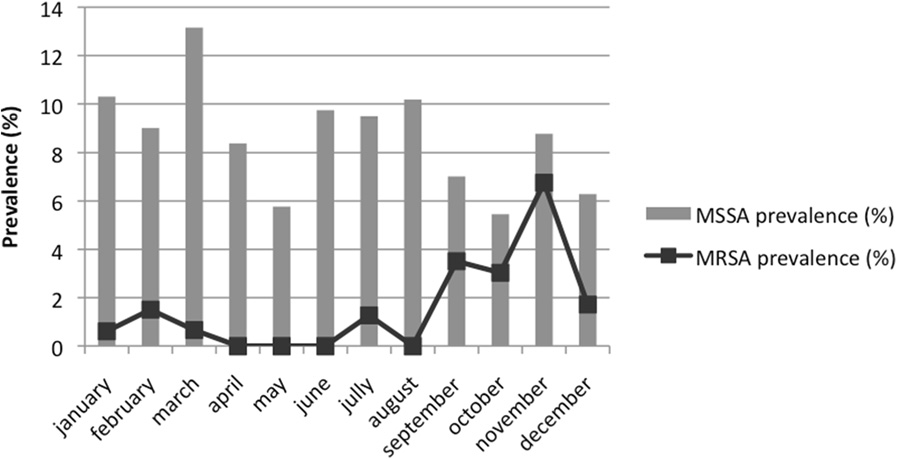
MSSA and MRSA monthly prevalence in 2009 for NCC.

Distribution of the different types of SA positive clinical samples corresponding to colonization and/or infection by care sector is summarized in Table 1. Most of them occurred in the PRICU (70.9% of all positive samples), followed by the NICU (23.3%) and the kangaroo ward (5.8%). Respiratory samples were predominant in the NCC, overall representing 52.3% of all clinical samples, especially in the PRICU (63.9%). Ophthalmic samples were the second most frequent with about 17.5%, followed by blood cultures (10%) and catheter samples (7%). By care sector, ophthalmic colonization and infections predominated in the NICU, and came equal with respiratory samples (40% each) in the kangaroo ward. SA positive catheter samples and blood cultures were mainly present in the PRICU (8 of the 10 samples), 2 other cases were found in the NICU, and none in the kangaroo ward where patients are less often perfused.The evolution of SA monthly prevalence for each sector is represented on Figure 2. In the NICU, where digestive carriage of SA is routinely screened, average prevalence reached 12.9%, with increasing rates in March (20%), August (17%) and November (22%). In other sectors, because of the absence of systematic screening, prevalence rates obtained were certainly underestimated and did not reflect the real presence of SA. In the PRICU, average rates were around 5.5% and ranged generally between 5 and 10%, except in November and December when they fell to 3.1% and 1.1% respectively. In the kangaroo ward, rates were more variable, roughly around 6.2% with 2 peaks in June (16.2%) and November (14.3%). Monthly incidences of SA clinical samples and infections by care sector were also calculated. Evolution of incidences over the year was very similar to prevalence curves, especially in the NICU with increased incidence in March (26.1%), July (20%) and August (17.4%), and November (28.9%) (data not shown). Finally, epidemiological analyses showed several epidemic episodes on a background of SA endemicity: each care sector presented 2 or 3 prevalence peaks.Monthly MSSA and MRSA prevalence rates in the entire NCC are summarized in Figure 3. The overall MSSA prevalence did not decrease below 5.5% (in May and October), and reached 13.2% in March. Concerning MRSA, the mean prevalence in 2009 for all of the NCC was around 1.53%, with increasing rates in September (3.7%) and November (6%). These results show that MSSA endemicity largely exceeded MRSA endemicity in the NCC. Incidence rates confirmed that new cases of MSSA were also more frequent than MRSA in the NCC (data not shown).

**Table 1:** Typological analysis of colonization- and infection-associated clinical SA positive samples by care sector

	** NICU**		** PRICU**		** Kangaroo ward**		** Total**	
	**n**	**%**	**n**	**%**	**n**	**%**	**n**	**%**
Sites of isolation								
Respiratory	4	20	39	63.9	2	40	45	52.3
Ophthalmic	9	45	4	6.6	2	40	15	17.5
Blood	2	10	8	13.1	0	0	10	11.6
Catheter	2	10	5	8.2	0	0	7	8.1
Other	3	15	5	8.2	1	20	9	10.5
Total	20		61		5		86	

Monthly analysis of infections showed an emergence of SA bacteraemia and catheter-linked infections in summer. For one of them, positive blood-culture was associated with a positive catheter. The origin of the other bacteraemia was not found.

### MSSA infections outbreak

**Figure 4:**
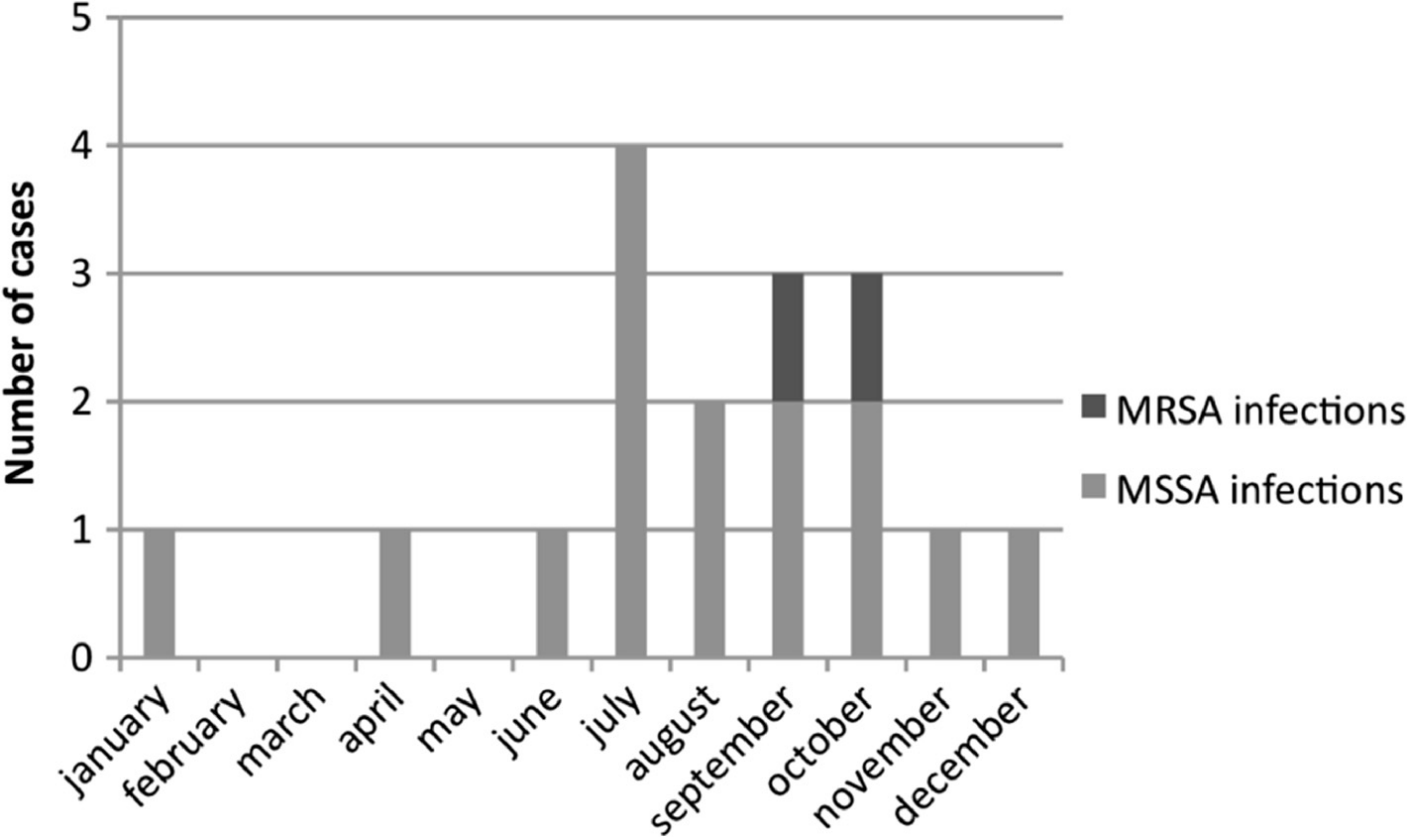
Evolution of SA infections in 2009 in NCC.

The epidemic curve of SA infection cases between January and December 2009 is shown in Figure 4. Among the 17 SA infections, 15 were caused by MSSA strains versus 2 by MRSA. Outbreak alert was sounded because of an increase in MSSA case numbers observed in July (4 cases) and August (2 cases), contrasting with the average rate of one case every 2 months until then. Six other SA infections occurred in September and October with respectively 3 cases including one MRSA infection each month. Another MSSA infection in November and one in December were also reported.

The 6 cases of July and August led to the outbreak investigation. All 6 patients were hospitalized in the PRICU and 5 of them were preterm neonates. Principal characteristics of these patients and associated strains are given in Table 2. Antimicrobial susceptibility was tested for the 12 MSSA strains from the 6 patients (Table 2). The 12 strains were mostly penicillinase producers (n = 11) and susceptible to most antibiotic classes except one strain resistant to macrolides. Most strains lacked virulence factors since only one carried *tst*, the gene encoding for toxic shock syndrome toxin 1 and one other carried *sea* coding for enterotoxin A. PFGE-typing for the 12 strains revealed a predominant profile named pulsotype C and shared by 8 strains isolated from 4 patients (Table 2). Interestingly, some isolates with the same pulsotype displayed different content in virulence genes.

**Table 2:** Principal clinical characteristics of outbreak patients and strains

**Patient**	**Reason of admission**	**Samples**	**Nature of sample**	**NCC sector**	**PFGE profile**	**MSSA/MRSA**	***luk-PV***	***sea***	***tst***	**Penicilline G**	**Oxacilline**	**Kanamycin**	**Gentamicin**	**Tobramycin**	**Tetracycline**	**Macrolides**	**Cotrimoxazole**	**Fluoroquinolones**	**Rifamycin**	**Fusic acid**	**Fosfomycin**	**Glycopeptides**	**Linezolid**
1	Prematurity and NEC	1A	BSI	NICU	A	MSSA				S	S	S	S	S	S	S	S	S	S	S	S	S	S
		1B		PRICU	B	MSSA				R	S	S	S	S	S	S	S	S	S	S	S	S	ND
2	Severe epileptic encephalopathy	2A	BSI	PRICU	C	MSSA				R	S	S	S	S	S	S	S	S	S	S	S	S	S
		2B	Conjunctivitis		C	MSSA		+		R	S	S	S	S	S	S	S	S	S	S	S	S	S
3	Prematurity and NEC	3A	Insertion point of KT	PRICU	C	MSSA				R	S	S	S	S	S	S	S	S	S	S	S	S	ND
		3B	KT		C	MSSA	ND	ND	ND	R	S	S	S	S	S	S	S	S	S	S	S	S	ND
		3C			C	MSSA	ND	ND	ND	R	S	S	S	S	S	S	S	S	S	S	S	S	ND
4	Prematurity	4A	BSI	PRICU	Untypable	MSSA				R	S	S	S	S	S	S	S	S	S	S	S	S	S
		4B			Untypable	MSSA	ND	ND	ND	R	S	S	S	S	S	S	S	S	S	S	S	S	S
5	Prematurity	5A	Tracheal Asp.	PRICU	C	MSSA				R	S	S	S	S	S	S	S	S	S	S	S	S	ND
		5B	BL		C	MSSA			+	R	S	S	S	S	S	R	S	S	S	S	S	S	S
6	Severe epileptic encephalopathy	6A	Tracheal Asp.	PRICU	C	MSSA			-	R	S	S	S	S	S	S	S	S	S	S	S	S	S

NEC: necrotizing enterocolitis, BSI: bloodstream infection, KT: catheter, Asp.: aspiration, BL: bronchial liquid, *luk-PV*, gene coding for Panton-Valentine leukocidin; *sea*, genes coding for staphylococcal enterotoxins A; *tst*, gene coding for toxic shock syndrome toxin 1, ND, non determinate.

The synopsis of the IC team’s interventions and investigations is presented in Table 3. During the environmental investigation, a high level of surface contamination (109 surfaces sampled) in all areas (average rate of 82.6%) of the NCC was observed with the presence of pathogens such as *Enterococcus faecalis*, *Pseudomonas aeruginosa* and MSSA. MSSA were isolated from a rack of electric syringes, medical records of patient, 2 spare beds stored in the hallway, and an incubator. All of these environmental MSSA strains were typed by PFGE and differed from the profile C. Water samples (n = 19) at various points of use were also analyzed, but none was positive for SA.

**Table 3:** Outbreak investigation and IC interventions

**Actions**	**Dates**
Case study	August 07, 17 and 18, 2009
- Clinical records review and strains typing	
Environmental investigation	August 05, 12, September 15 and October 13, 2009
- Surface and water sampling	
- Gowns	December 2009 (retrospectively)
Hygiene practices assessment	October 01, 2009
- Hand hygiene	October 19, 24 and November 09, 2009
- Catheter-linked practices	

The clinical audit of hand hygiene practices revealed compliance rates of 60% in the NICU (14 persons assessed) and 76% in the PRICU (16 persons assessed). Non-compliance with recommendations was mostly due to the concatenation of multiple care sequences for the same patient, for example the absence of hand hygiene between a contaminated care (nap change) and a clean one (catheter manipulation). The assessment of central venous catheter manipulations in the PRICU revealed failures in catheter monitoring: catheter insertion points were covered with opaque dressings preventing visual control, and the frequency of dressing changes was insufficient. Furthermore, health-care workers always wore white over-gowns whenever tending to a catheter or coming into direct contact with perfused patients. These gowns were stored in a drawer of the incubator until re-use. During the observations, these white gowns were pointed out as a potential bacterial reservoir or source of cross-contamination. Thereby, 35 of them were sampled in December: 25 gowns in the PRICU and 10 in the NICU. Many of them were positive (15/35) for several pathogens including SA, which was found on 8 separate gowns. Overall these gowns showed a high level of contamination (on average 50 CFU/25 cm^2^). SA strains were not typed due to the delay since the summer outbreak but the level of contamination suggested that misuse of these gowns could promote the transmission of pathogens.

## Discussion and conclusion

The study of SA epidemiology in the Montpellier NCC showed an MSSA infection outbreak in the context of SA endemicity. The overall prevalence of SA in 2009 was consistent with other published studies [11,21]. The SA outbreak consisted mostly of bloodstream and respiratory tract infections, mainly caused by the same clone in PFGE. Observations by the IC team suggested that slack healthcare practices could be directly linked to the outbreak. Poor practice concerned mainly the manipulation and monitoring of central venous catheters, and the use of non-disposable white gowns. Aside from constant attention to hand hygiene [22,23], the complexity of care in neonatology requires a perfect knowledge of infection control principles, enabling healthcare professionals to cluster interventions by risk level so as to limit self-contamination of the patient or contamination of his environment [22,23]. Some studies have confirmed the progressive contamination of hands or gloves [24] and identified key opportunities for hand hygiene during routine cares, even if wearing gloves, because of possible hand contamination during removal [22]. Moreover, health care workers’ white coats remain a controversial subject, supposedly protecting the patient during central venous catheter manipulations, but highly contaminated by pathogens for most of them (about a quarter positive for SA). As previously described by Treakle *et al.* in 2009, we could imagine that they were a vector of patient-to-patient transmission, relaying SA circulation and outbreak in the NCC [25]. However, we did not investigate the possibility of SA chronic carriage by a health care worker which could also have relayed the outbreak [8,26].

The typological analysis of colonized sites and infections occurring in 2009 showed a majority of cases in the PRICU, where patients are most susceptible to infections. The occurrence of catheter-related infections in the summer was consistent with the increased SA prevalence (colonization pressure), and slack catheter monitoring. Catheter-related infections are the most common health-care associated infections in NICUs [22,27,28]. The general strategy for their prevention is based on good practice recommendations concerning (i) insertion and maintenance of indwelling lines, (ii) administration of prophylactic antibiotics e.g.: antibiotic lock therapy, (iii) use of skin emollients to reduce bacterial penetration, and (iv) health-care workers and visitors donning of single-use gowns [22]. However in our NCC, points (ii) and (iii) were not applied and compliance with points (i) and (iv) was not optimal.

SA is a significant pathogen in neonatology and an important cause of morbidity [7]. Epidemiological studies of MRSA in NICUs have reported widely varying prevalence rates, ranging from 0.6 to 53% [6,7,21,29,30], and MRSA outbreaks are often described [6,31]. Far less attention is given to MSSA. In the Montpellier NCC, MRSA prevalence appeared rather low (1.53% in 2009, ranging from 0 to 6.1%), and quantitatively a less important problem than MSSA. We believe the commonly encouraged focus on MRSA surveillance [22,27,28] may lead to unrecognized or underestimated spread of MSSA. This is particularly worrying for infection control in wards where MSSA is the most prevalent SA type, as we observed for the NCC.

## Abbreviations

CoNS: Coagulase-negative staphylococci; HAI: Health-care associated infections; IC: Infection control; *luk- PV*: gene encoding the panton-valentine leukocidin; MDRB: Multi-drug resistant bacteria; MRSA: Methicillin-resistant *Staphylococcus aureus*; MSSA: Methicillin-susceptible *Staphylococcus aureus*; NCC: Neonatal care center; NICU: Neonatal intensive care unit; PFGE: Pulsed-field gel electrophoresis; PRICU: Pediatric reanimation and intensive care unit; PVL: Panton-valentine leukocidin; SA: *Staphylococcus aureus*; *sea* gene: Encoding the staphylococcal enterotoxins A; *tst* gene: Encoding the toxic shock syndrome toxin 1.

## Competing interests

The authors declare that they have no competing interests.

## Authors’ contributions

SRB, AF, RM, MND and GC participated in the data collection. SRB and AF contributed to bacteriological and epidemiologic analysis. SRB, AL, LB, EJB and SP contributed to outbreak investigation and data interpretation. SRB and AF wrote the manuscript. All authors read and approved the final manuscript.
